# Harnessing the mTOR Pathway for Tuberculosis Treatment

**DOI:** 10.3389/fmicb.2018.00070

**Published:** 2018-01-30

**Authors:** Pooja Singh, Selvakumar Subbian

**Affiliations:** Public Health Research Institute at New Jersey Medical School, Rutgers Biomedical and Health Sciences Rutgers, The State University of New Jersey, Newark, NJ, United States

**Keywords:** mTOR, autophagy, everolimus, host directed therapy, tuberculosis, drug resistance, adjunct therapy, phagocytosis

## Abstract

Tuberculosis (TB) remains as one of the leading killer infectious diseases of humans. At present, the standard therapeutic regimen to treat TB comprised of multiple antibiotics administered for a minimum of six months. Although these drugs are useful in controlling TB burden globally, they have not eliminated the disease. In addition, the lengthy duration of treatment with multiple drugs contributes to patient non-compliance that can result in the development of drug resistant strains (MDR and XDR) of Mycobacterium tuberculosis (Mtb), the causative agent of TB. Therefore, new and improved therapeutic strategies are urgently needed for effective control of TB worldwide. The intracellular survival of Mtb is regarded as a cumulative effect of the host immune response and the bacterial ability to resist or subvert this response. When the host innate defensive system is manipulated by Mtb for its survival and dissemination, the host develops disease conditions that are hard to overcome. The host intrinsic factors also contributes to the poor efficacy of anti-mycobacterial drugs and to the emergence of drug resistance. Hence, strengthening the immune repertoire involved in combating Mtb through host-directed therapeutics (HDT) can be one of the approaches for effective bacterial killing and clearance of infection/disease. Recently, more scientific research has been focused toward HDT strategies that empowers host cells for effective killing of Mtb, reduce the duration of treatment and/or alleviates the development of MDR/XDR, since Mtb cannot develop resistance against a drug that targets the host cell function. Autophagy is a conserved cellular process critical for maintaining cellular integrity and function. Autophagy is regulated by multiple pathways that are either dependent or independent of mTOR (mechanistic target of rapamycin; a.k.a. mammalian target of rapamycin), a master regulatory molecules that impacts several cellular functions. In this review, we summarize the role of autophagy in Mtb pathogenesis, the mTOR pathway and, modulating the mTOR pathway with inhibitors as potential adjunctive HDT, in combination with standard anti-TB antibiotics, to improve the outcome of current TB treatment.

## Introduction

Tuberculosis (TB) is one of the leading killer among infectious diseases of humans, accounting for about 10.4 million new cases and 1.8 million deaths in 2015 (World Health Organization, 2016). The global burden of TB has also been exacerbated by other co-morbid conditions, including diabetes and HIV-infection, and TB is a leading cause of mortality among HIV infected individuals with nearly 400,000 deaths reported in 2015. The standard therapeutic regimen recommended by the WHO for treating drug-sensitive pulmonary TB, known as DOTS (Directly Observed Treatment, Short course), is comprised of four antibiotics: isoniazid (INH), rifampicin (RIF), pyrazinamide (PZA) and ethambutol (ETH) for 2 months (initial phase) followed by INH, and RIF for 4 months (continuation phase). This multi-drug regimen is essential and necessary to ensure successful bacteriological cure in patients with TB. Although these drugs are useful in controlling the overall disease burden at the level of individual patients as well as global TB control measures, they have not eliminated the disease at both these levels (Ryan, 1992). This is in part due to the lengthy duration of treatment with multiple drugs, which promotes the fear of drug dependency and doubts of not getting cured and contributes to drug-induced tissue toxicity issues. Adverse effects, ranging from serious ones, like hepatitis and pneumonia, to minor ones, like vomiting, acne and nausea, have been reported to be associated with DOTs therapy (Michael et al., 2016). Thus, high drop-out rate of TB patients from treatment regimens (a.k.a. patient non-compliance) is a serious issue contributing directly to the development of drug resistance in *Mycobacterium tuberculosis* (Mtb), the causative agent of TB.

Development of drug resistance in a single bacterium has been suggested to be sufficient to create an outbreak of drug resistant bacteria (Borrell and Gagneux, 2009). In 2015, nearly 4.8 million cases of isoniazid- and rifampicin-resistant [a.k.a. multidrug-resistant TB (MDR-TB)] cases were reported. In addition to INH and RIF (the first line drugs), Mtb can develop resistance to PZA and ETH (second line drugs) and other injectable aminoglycosides, leading to extensively drug-resistant TB (XDR-TB) cases. Nearly 9.5 % of all MDR-TB cases in 2015 were estimated to be XDR-TB. A recent study aimed at predicting the future burden of TB suggests an increased prevalence of MDR and XDR cases due mainly to person-to-person transmission of drug-resistant Mtb, rather than the pathogen acquiring drug resistance within the infected host (Sharma et al., 2017). Hence, current treatment strategies demand intense patient monitoring during and after drug treatment, which poses major strategical and economical challenges for the global TB control programs conducted by various health agencies. Therefore, it is imperative that new anti-TB therapies are developed and implemented to shorten the number of antibiotics taken and/or duration of treatment, to lower the drug- induced toxicities, and to improve the drug efficacy among TB patients with co-morbid conditions, such as HIV-infection and/or patients with MDR/XDR-TB.

Development of drug resistance among infecting Mtb is also dependent on host intrinsic factors, such as genetic make-up, health, and well-being, all of which impact the immune response against the bacteria. A key component of the host innate defense system are macrophages, phagocytic cells that engulf and destroy infecting microorganisms. However, Mtb can “invade” macrophages (and other host cells), where it is able to survive, proliferate and cause infection/disease. Invasion of macrophages by Mtb brings changes to the normal phagocytosis events, such as calcium ion homeostasis, membrane protein distribution and phagosome-lysosome fusion. If/when Mtb survives, it continues to multiply intracellularly and induce a pro-inflammatory response, leading to the onset of cell mediated/adaptive immunity and granuloma formation, which is generally thought of as a region of equilibrium between the host and the bacterium. For Mtb, the granuloma serves as an environment where the bacteria can exist in a dormant, semi-and/or non-replicating state. For the host, the granulomas restrict the spread of Mtb to other tissues/organs because the diseased area is cordoned-off by the activated immune cells (Guirado et al., 2013). The host-pathogen interactions in the granuloma are highly complex, where the bacteria may get killed or able to survive and persist (Flynn and Chan, 2003). Taken together, the intracellular survival of Mtb is regarded as a cumulative effect of the host immune response and the bacterial ability to resist or subvert this response. Hence, strengthening the immune repertoire involved in combating Mtb through host-directed therapeutics (HDT) can be one of the approaches for effective bacterial killing and clearance of infection/disease.

**Figure 1:**
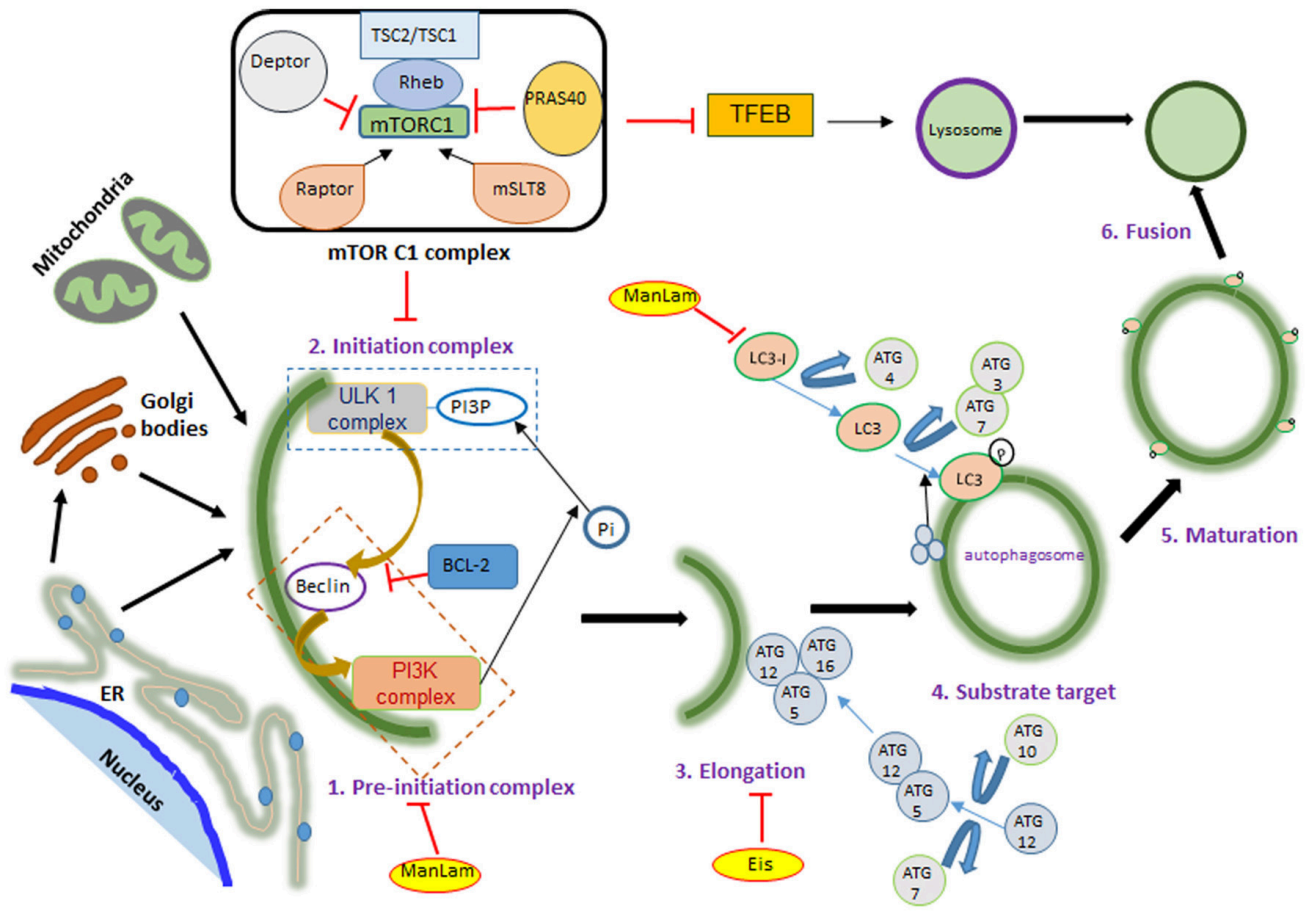
mTOR signaling during autophagy. mTOR has two complexes: mTORC1 and mTORC2, differentiated according to their activator proteins Raptor and Rictor. When activated, mTOR blocks ULK complex formation by phosphorylating it. ULK and PI3K complex formation marks the initiation of autophagy, followed by ATG-5, 12, and 16 binding on the phagophore membrane. This leads to LC3 translocation on the autophagosome membrane, which is required for fusion with lysosome and ultimately bacterial killing. Autophagy activating factors and pathways are denoted in black color and autophagy inhibitors are in red color letters and lines; mTOR activation leads to protein synthesis, nucleotide synthesis, cytoskeletal regulation and ion transport. These are denoted in blue color. Yellow color highlight denotes mycobacterial factors influencing autophagy.

Host directed therapy (HDT) aims at manipulating the metabolism and/or immune cell function to optimize the pro-inflammatory response or to modify the tissue physiology (Subbian et al., 2011a,b; Tobin et al., 2012). Recently, research on HDT as potential therapeutic strategy for infectious diseases has gained significant momentum due to the possibility of re-purposing drugs that have been already approved to treat chronic ailments and the advantage that pathogenic bacteria, such as Mtb, cannot develop resistance against a HDT, which targets host cell functions (Zumla et al., 2015). Autophagy is a homeostatic cellular process that removes intracellular debris derived from endo-and exo-genous sources, thus ensuring efficient functioning of cells. It is also a key innate immune response of the host cells to protect against invading pathogens. Therefore, targeting the autophagy machinery using small molecules and drugs to improve the host cell effector functions is an emerging concept in the treatment of several chronic diseases (Rubinsztein et al., 2012). Autophagy is regulated by multiple, complex networks and pathways that are either dependent or independent of mTOR (mechanistic target of rapamycin; a.k.a. mammalian target of rapamycin), a master regulatory molecule that impacts several cellular functions (Figure 1). In this review, we focus mainly on the role of autophagy in Mtb pathogenesis and modulating the mTOR pathway as potential adjunctive HDT to improve current, antibiotic-based treatment for pulmonary TB.

## Modulation of phagocyte function by *Mycobacterium tuberculosis*

Successful intracellular pathogens inhibit host cell antimicrobial processes involved in restricting their survival (Flynn and Chan, 2003; Kim et al., 2012). In that context, Mtb is known to inhibit killing within the phagolysosome of macrophages and other antigen presenting cells (APC) by modulating phagosome maturation and its fusion with the lysosome. In the infected APC, pathogenic Mtb inhibits actin assembly around the phagosome, thereby inhibiting host lipid molecules from interacting with phagosomal proteins necessary for further maturation and fusion with the lysosome (Vergne et al., 2003; Rohde et al., 2007; Ehrt and Schnappinger, 2009; Shui et al., 2011; Seto et al., 2012). When bacteria are phagocytosed by APC, the phagosome acquires early endosomal protein markers, such as EEA1 (early endosomal antigen 1) and Rab5, which are gradually replaced with Rab7 during the maturation of the phagosome (Chandra et al., 2015); ultimately, LAMP1 (lysosome-associated membrane protein 1) and acid hydrolases mark the late phagosome for fusion with lysosome (Huynh et al., 2007). It has been reported that phagosomes containing live Mtb do not acquire Rab5 due to the presence of tryptophan aspartate coat protein (TACO). Phagosomal association with TACO is also reported in macrophages that can engulf other pathogenic mycobacteria, which also result in the inhibition of phagosomal maturation (Pieters and Gatfield, 2002). Proper maturation of phagosomes is the key to its fusion with lysosomes, which can kill the bacteria by delivering toxic molecules. However, due to the absence of proton-ATPase molecules in Mtb-containing phagosomes, the phagosome-lysosome fusion does not take place and the bacteria survive intracellularly (Vergne et al., 2005).

Another mechanism used by Mtb to manipulate APC involves perturbation of intracellular calcium ion (Ca^2+^) levels (Kusner and Barton, 2001). Several studies have demonstrated fluctuations in intracellular Ca^2+^ levels in Mtb-infected macrophages (Vergne et al., 2003; Jayachandran et al., 2007). During phagocytosis of opsonized or heat-killed Mtb, intracellular Ca^2+^ concentrations increase, while macrophages infected with live pathogenic Mtb have reduced calcium ion level, which in turn significantly reduce the levels of Ca^2+^ associated-calmodulin and the phosphorylated Ca^2+^/calmodulin-dependent protein kinase II (CaMKII) (Jayachandran et al., 2007). Reduction in CaKMII level also blocks the delivery of lysosomal components to phagosome. Mtb reportedly prevents intracellular Ca^2+^ increase through its cell wall glycolipid, ManLAM (mannose-capped lipoarabinomannan), and by inhibiting host sphingosine kinase (SK). ManLAM also inhibits ionophore-induced increase in Ca^2+^ levels in macrophages. Reduced Ca^2+^/Calmodulin association impairs PI3K signaling, which inhibits recruitment of EEA1 to phagosomes (Rojas et al., 2000). Inhibition of SK abrogates phosphorylation of sphingosine, which is required for G-protein coupled receptor (GPCR) signaling that regulates Ca^2+^ homeostasis. Thus, ManLAM triggers a sequence of events leading to Ca^2+^ signaling disruption and phagosomal maturation arrest, which facilitate successful intracellular survival of infecting Mtb (Chan et al., 1991; Rojas et al., 2000).

Apart from its function in maintaining cellular homeostasis, autophagy is also known to sense and destroy intracellular bacteria in innate immune cells, such as macrophages. Although intracellular Mtb can efficiently modulate the bactericidal mechanisms of phagocytes, autophagy has been shown to be effective in killing Mtb (Gutierrez et al., 2004; Maiuri et al., 2007). Xenophagy, a type of autophagy whereby microorganisms can be sequestered and subject to lysosomal degradation, has been proposed to play an important role in elimination of bacteria (Gutierrez et al., 2004; Rubinsztein et al., 2012; Songane et al., 2012). In Mtb-infected host cells, the autophagosome collects ubiquitin while maturing, which then ultimately fuses with lysosome, thereby enhancing the lysosome-mediated bacterial killing. Survival of Mtb in macrophages has been reported to be dependent on the autophagosome delivery to the lysosome. However, *in vivo* and *in vitro* results have shown disparity in Mtb survival following inhibition of autophagy markers (Table 1) (Levine and Deretic, 2007; Lerena et al., 2008; Levine et al., 2011). Mutation or knockdown of autophagy associated host genes, such as Unc-51-like kinase 1 (Ulk1), Beclin1, Atg5, Atg7 or p62, has been reported to increase the survival of intracellular bacteria (Kim et al., 2011; Mizushima et al., 2011; Shang et al., 2011; Alers et al., 2012). However, although xenophagy is reported to restrict the survival of Mtb and BCG (bacille Calmette-Guerin) within macrophages, there are studies suggesting that intracellular pathogens such as *Shigella flexneri, Listeria monocytogenes, Burkholderia pseudomallei, Orientia tsutsugamushi, Porphyromonas gingivalis, Staphylococcus, Brucella abortus*, and *Salmonella typhimurium* are capable of blocking induction of autophagy by downregulating the co-localization of LC3 (microtubule-associated protein 1A/1B-light chain 3), restoring activation of mTOR, and utilizing nutrients for their growth and survival (Thurston et al., 2009; Yoshikawa et al., 2009; Zheng et al., 2009; Choy et al., 2012; Fraunholz and Sinha, 2012; Asrat et al., 2014; Yu et al., 2014).

**Table 1:** Major differences and similarities between mTOR complexes- mTORC1 and mTORC2.

**Similarities**	**Differences**	
	**mTOR C1**	**mTOR C2**
•Both belong to mTOR signaling cascade. •Member of PI3K related kinase family. •mLST8 is a common positive regulator protein. •Cellular stress, such as low level of growth factors, generation of reactive oxygen species and energy depletion inhibits mTOR signaling.	Five components: mTOR, RAPTOR, mLST8, PRAS40, and DEPTOR.	Six components: mTOR, RICTOR, DEPTOR, mSIN-1, mLST8, and PROCTOR.
	Positive regulators: RAPTOR and mLST8.	Positive regulators: RICTOR and mSIN-1.
	Negative regulators; PRAS40 and DEPTOR.	Negative regulators: DEPTOR.
	Rapamycin sensitive.	Rapamycin insensitive.
	Inhibits autophagy by directly interacting with pre initiation complex (ULK complex).	Inhibits autophagy indirectly by regulating mTORC1.
	Regulates p70-S6K and 4E-BP-1 to influence cellular metabolism.	Regulates AKT to influence cellular growth.
	Activating signaling: Growth factors, energy molecules, amino acids level.	Not known.

Pathogenic Mtb also inhibits phagosome function in infected macrophages by releasing vesicle-bound lipids and glycolipids, which accumulate in lysosomes and interfere with the phagosome-lysosome fusion (Beatty et al., 2000). Taken together, pathogenic intracellular Mtb uses multiple strategies to manipulate the host defense machinery of APC for its own survival. Manipulation of APC function by Mtb impacts subsequent downstream events, including autophagy, antigen presentation, apoptosis, and activation of various signaling pathways involved in the production of cytokines, chemokines and other effector molecules that are crucial for controlling bacterial growth and replication (Briken et al., 2004; Cooper, 2009; Guenin et al., 2009; Rajaram et al., 2010).

## Autophagy and mTOR signaling

Autophagy, a Greek word meaning “eating of self,” is a conserved cellular process critical for maintaining cellular integrity and function. This catabolic process is activated in cells due to lack of nutrient availability or cellular damage or stress, and involves degradation of damaged organelles and misfolded or abnormal proteins. During starvation, cytosolic components of cells are sequestered by autophagy to release nutrients for de novo biosynthesis of molecules (Laplante and Sabatini, 2012). Autophagy can also be activated by pathological factors, such as infections and other diseases. In these cases, normal cellular functions are facilitated by the elimination of pathogens through autophagy-dependent mechanisms, such as surface antigen presentation (Rubinsztein et al., 2012; Songane et al., 2012). Moreover, autophagy is one of the macrophage defense mechanisms against Mtb infection.

Autophagy is characterized by phagophore formation, elongation and maturation of the autophagosome, which ultimately fuse with the lysosome for the degradation of its contents. Formation of the autophagosome begins with a double membranous structure derived from the lipid bilayers of the endoplasmic reticulum (ER) or Golgi apparatus and conjugated with autophagy related (ATG) proteins (Maiuri et al., 2007; Alers et al., 2012). The three main components of autophagosome generation are: PI3KC3 (class III phosphoinositide 3-kinase complex 3), ULK1 (unc-51-like kinase 1) complex and ATG complex (Figure 1). This process is negatively regulated by mTOR kinase, which, when activated, blocks the ULK1 complex (Kim et al., 2011; Shang et al., 2011). Under stress conditions, such as nutrient deprivation or bacterial invasion, mTOR gets inactivated, enabling the ULK1 complex to recruit and activate PI3KC3 (Dibble and Cantley, 2015). This initiation complex, formed on the ER, leads to the nucleation of cell membrane, which is followed by recruitment of an ubiquitin-like molecule, LC3. In the final step, LC3 conjugates with phosphotidylethanolamine, resulting in self-fusion of the double membrane to form the autophagosome, which subsequently fuses with lysosome to degrade the engulfed contents.

Apart from mTOR signaling pathway (Lipinski et al., 2010), autophagy is also regulated by the inositol signaling pathway (Sarkar et al., 2005), Ca^2+^ /Calpain signaling pathway (Gordon et al., 1993) and cAMP (cyclic adenosine monophosphate) (Noda and Ohsumi, 1998). Inhibition of these mTOR-independent pathways for promoting autophagy has been studied under different disease conditions (Floto et al., 2007; Grumati et al., 2010; Hidvegi et al., 2010). Promising outcomes of autophagy induction via mTOR-independent pathway have been observed only with a combination therapy strategy, where the small molecules enhancers (SMERs) or inhibitors (SMIRs) of mTOR-independent pathway are used in combination with an mTOR inhibitor. For example, lithium, an inositol (1,4,5)-triphosphate inhibitor, when administered with rapamycin results in a stronger induction of autophagy (Sarkar et al., 2008). On the other hand, rapamycin alone can induce autophagy even at high intracellular inositol (1,4,5)-triphosphate levels, which has autophagy inhibitory effects. Since targeting ULK complex formation or ATG complex, rather than affecting the upstream pathways, seems to have a specific and stronger impact on autophagy, mTOR has been the target of interest for promoting autophagy upon infection with *Mycobacteria* (Gutierrez et al., 2004).

## The mTOR complex

**Figure 2:**
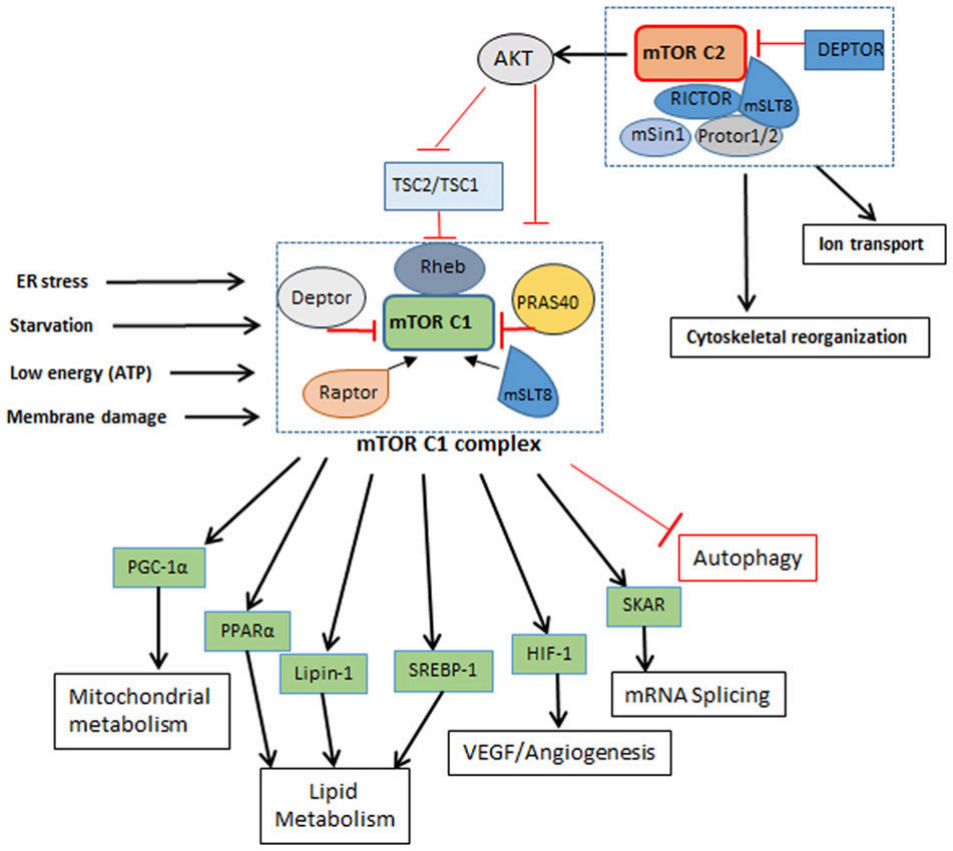
mTORC1 and mTORC2 complexes. mTORC1: Is a five component complex with DEPTOR and PRAS40 as negative regulators and RAPTOR and mSLT8 as positive regulators. It regulates different cellular processes like lipid metabolism and protein metabolism other than autophagy as seen in Figure 1. It is influenced by the nutrient and energy level in cell and gets shut down or inhibited when cell encounters reducing nutrient level and decrease in energy. Inhibition of mTORC1 leads to inhibition of cellular metabolic processes. mTORC2; A six component complex has DEPTOR as its negative regulator and RICTOR and mSLT8 as positive regulators. This complex influences activation of mTORC1 by phosphorylation of AKT. How nutrient level influences mTORC2 is not known yet.

In addition to its role in autophagy, mTOR is also a master regulator of cell metabolism, growth, proliferation, translation initiation, and cytoskeletal organization. It belongs to the family of phosphoinoside 3-kinase- (PI3K-) related kinase and is a highly conserved serine/threonine protein kinase, which exists in host cells as part of two protein complexes—mTORC1 and mTORC2 (Laplante and Sabatini, 2012; Singh and Cuervo, 2012) (Figure 2). Theses complexes differ in their structure and activity, in part due to the difference in mTOR regulatory proteins such as RAPTOR (regulatory associated protein of mTOR; rapamycin sensitive) in mTORC1 and RICTOR (rapamycin-insensitive companion of mTOR) in mTORC2, as well as other accessory proteins (Laplante and Sabatini, 2009). The proteins that are common to both mTORC1 and C2 complexes are the mammalian lethal with Sec13 protein 8 (mLST8) and DEP domain containing mTOR interacting protein (DEPTOR). While mLST8 acts as a positive regulator, DEPTOR functions as a negative regulator of mTOR signaling. The mTORC1 is activated by RAPTOR, PRAS40 (proline-rich AKT substrate 40 kDa) and by phosphorylation of tuberous sclerosis protein 2 (TSC2) (Huang et al., 2008). The PI3K/AKT pathway is a positive regulator of mTOR signaling (Kim et al., 2011; Ng et al., 2011; Pan et al., 2012). Apart from PI3K/AKT, arginine, DNA damage, AMPK (AMP-activated protein kinase) and ERK1/2 (extracellular signal-regulated protein kinases 1 and 2) signaling were also reported to regulate mTORC1 activation (Kim et al., 2002; Inoki et al., 2003; Fingar and Blenis, 2004; Laplante and Sabatini, 2009). Importantly, mTORC1 activation inhibits autophagy (Jung et al., 2010). Deactivation of mTORC1 in cells under nutrition depletion or treatment with rapamycin leads to initiation of autophagy. (Seto et al., 2013). Similarly, dephosphorylating ULK1 by inactivation of mTORC1 induces autophagy (Egan et al., 2011; Kim et al., 2011; Shang et al., 2011).

The binding of mTORC2 with RICTOR facilitates the interaction of these proteins with TSC2 and mammalian stress-activated protein kinase interacting protein (mSIN-1); another protein found in association with RICTOR is PROTOR-1, which promotes activation of serum and glucorticoid-induced kinase 1 (SGK1). Interaction of all these proteins ultimately promotes mTORC2 complex formation and phosphorylation of AKT. Therefore, mTORC2 activation also regulates mTORC1 activation via AKT phosphorylation. Similarly, while mTORC1 activation is mediated by PI3K-AKT/PKB pathway in response to nutrient availability and mitogenic stimulation of the cell, phosphorylation of growth factors by autophosphorylation of their receptor tyrosine kinases activates mTORC2 complex, which also activates class I PI3K-AKT/PKB pathway (Dibble and Cantley, 2015). mTOR complexes also differ in the nature of their stimulant, for example, mTORC1 is activated by low levels of amino acids and growth factors, energy molecules and stress, while mTORC2 remains unaffected by the changing levels of these mTORC1 stimulants. However, role of mTORC2 is important for the regulation of AKT, which in turn governs mTORC1 functions. With use of TSC deficient cells importance of autophagy for cell survival was validated. In conditions like TSC (tuberous sclerosis complex), mTOR inhibition by rapamycin and pro-survival due to autophagy may have beneficial effects (Parkhitko et al., 2011).

In addition to regulating autophagy, activation of mTORC1 also promotes cellular metabolic pathways, such as glucose metabolism, protein and lipid synthesis, all of which contributes to cell growth and proliferation. S6Ks (p70 ribosomal protein S6 kinase 1/2) and 4E-BPs (eukaryotic initiation factor4 binding protein) are the two major proteins interacting with mTORC1 and play a major role in protein synthesis. mTORC1 phosphorylates 4E-BP1 thereby inhibiting its interaction with elF4E, which is then able to promote cap-dependent translation. Similarly, mTORC1 interaction with S6K1 stimulates cap-dependent translation of ribosomal proteins. Phosphorylation of S6K by mTORC1 also activates glucose transporter protein (Glut1) which activates glycolysis, lipogenesis and increases glucose uptake (Zeng et al., 2016). This increased glycolysis due to Glut1 is also reported to elevate T cell function and proliferation (Macintyre et al., 2014). Likewise, lipid synthesis is influenced by positive regulation of SREBP1 (sterol regulatory element binding protein 1) and PPARγ (peroxisome proliferator-activated receptor-γ) (Kim and Chen, 2004), which are regulated by mTORC1. The mTOR inhibitor, rapamycin, reduced phosphatidic acid phosphatase (lipin-1) phosphorylation, which is essential for glycerolipid synthesis; lipin-1 also activates PPARγ and other proteins associated with lipid synthesis (Huffman et al., 2002). Oxidative metabolism is also influenced by mTOR signaling. In a mouse model, inhibition of mTORC1 reduced the muscle mass and oxidative metabolism, leading to early death. It has been shown that PGC1-α is associated with the oxidative metabolism and that mTOR directly interacts with this regulatory protein (Laplante and Sabatini, 2012). Other proteins interacting with mTORC1 are HIF-1α (hypoxia-inducible factor 1-alpha) and STAT3 (signal transducer and activator of transcription 3), which are involved in a plethora of cellular functions, ranging from angiogenesis to inflammation and cytokine response (Laughner et al., 2001).

## mTOR inhibitors as potential HDT for TB

Since mTOR signaling pathway regulates several cellular processes, including autophagy, that are linked to the host immune response to pathogens, it is an attractive target for developing/testing small molecules to modulate host immunity for better protection against infectious agents. Moreover, the peripheral blood mononuclear cells (PBMCs) and CD^4+^CD^25+^FoxP^3+^Treg cells isolated from active tuberculosis patients demonstrated mTOR inhibition during infection (Zhang et al., 2017). In contrast, mTOR activation, by deletion of *Tsc1* in hematopoietic stem cells, induces accumulation CDK (cyclin-dependent kinase) inhibitors p16^ink4a^, p19^Arf^, p21^Cip1^ leading to impaired hematopoietic system and decreased lymphopoiesis (Chen et al., 2009). These observations establish that mTOR inhibition improves cell survival and the understanding that mTOR inhibition may be promoting host cell defense mechanisms against invading pathogens (Harrison et al., 2009).

The following are some of the key mTOR inhibitors in use to treat chronic conditions in humans.

### Rapamycin

Rapamycin, specifically known for its mTOR inhibitory activity, was first isolated from *Streptomyces hygroscopicus*. Despite of its antifungal and antibacterial properties, Rapamycin is well-known for its immunosuppressant activity, which led to its use in organ transplant cases to reduce graft rejection. Similar to temsirolimus, rapamycin, which is also known as sirolimus, targets FKBP12 (FK506-binding protein 1A, 12 kDa) and inhibits the formation of active mTOR complex. Thus all of the currently known sirolimus derivatives target FKBP12 and inhibit mTOR complex.

In a zebrafish model of *M. marinum* infection, mTOR was shown to be associated with the host resistance to infection. In this model, mTOR mutants were hyper-susceptible to *M. marinum* at higher infection dose; however, when the inoculum size was decreased, the mTOR-deficient zebrafish cleared infection early (Pagan et al., 2016). Inhibition of mTOR in mice by rapamycin treatment at early age did not significantly affect the life expectancy or susceptibility to disease, but administration at an old age improved the survival expectancy (Harrison et al., 2009). In another study, administration of rapamycin to BCG-vaccinated mice has been shown to elicit better vaccination efficacy against Mtb infection, which is associated with induced autophagy, increased antigen presentation on dendritic cells and elevated Th1-type immune response (Gutierrez et al., 2004; Jagannath et al., 2009). Results from a low dose Mtb infection (MOI = 1) of human monocyte-derived-macrophages pre-infected with HIV, showed elevated bacterial load upon administration of rapamycin (1 μM) (Andersson et al., 2016). This study described mTOR inhibition as an advantage for the intracellular survival of Mtb; however, in an already immunocompromised cell (due to HIV infection), it is difficult to assess the impact of mTOR inhibition on Mtb growth. Although rapamycin used to be the popular drug of interest to achieve cellular mTOR inhibition, poor solubility and long intracellular half-life complicates the consideration of this molecule as potential HDT for TB therapy.

### Temsirolimus

Temsirolimus, commercially known as CCI-779 or Torisel, is currently approved by the US-FDA for use in renal cell carcinoma (RCC) treatment. This prodrug can transform to sirolimus when dihydroxymethyl propionic acid ester group at C40 position is removed. Temsirolimus is metabolized by the enzyme CYP3A4 (cytochrome P450 3A4) and has a half-life of 9–27 h (MacKeigan and Krueger, 2015). Intravenous administration of temsirolimus increases its bioavailability and dose intensity (Boni et al., 2009). Mechanistically, temsirolimus targets host FKBP-12 protein. The drug-FKBP-12 interaction inhibits the formation of mTOR-FKBP-12 complex, leading to the inactivation of mTOR complex and inhibition of p70S6k and S6 phosphorylation. These effects cumulatively results in arrested cell growth, proliferation and survival in RCC patients. Nonspecific pneumonitis and gastrointestinal disorders are major side effects in RCC patients treated with this drug. In addition, metabolic diseases such as hyperglycemia, and hypercholesterolemia are associated with temsirolimus administration in these patients (Malizzia and Hsu, 2008). Importantly, temsirolimus treatment has been widely associated with reactivation of latent Mtb infection among RCC patients. Also, progression of tumor was noted in these patients when temsirolimus was administered in combination with rifampicin, a first-line anti-TB drug (Bossé et al., 2016).

### Ridaforolimus

Ridaforolimus (AP23573 or MK-8669) is an analog of sirolimus with improved bioavailability, solubility and half-life (30–75 h) (Rivera et al., 2011). It is administered orally or intravenously for the treatment of solid tumors of soft tissues, bone and other hematologic malignancies (Huang et al., 2015). In a phase I clinical trial with 87 ER^+^/high-proliferative breast cancer cases, majority of patients treated with ridaforolimus demonstrated reduced tumor activity (Di Cosimo et al., 2015). Similarly, a phase II clinical trial showed promising results for ridaforolimus to treat patients with endometrial, soft tissue and bone cancers (Palavra et al., 2017). The effect of ridaforolimus on cell metabolism and growth is largely dependent on the dose of drug used for treatment. This is due to its varying effect on mTOR inhibition with variation in dosage (Rivera et al., 2011). However, no reactivation of latent Mtb infection has been reported in these studies (Huang et al., 2015; Palavra et al., 2017).

### Everolimus

Everolimus (40-O-(2-Hydroxy)-ethyl-rapamycin), commercially known as SDZ-RAD, RAD001, Certican and Afinitor, is a derivative of rapamycin bearing a stable 2-hydroxy ethyl chain substitution at position 40. This agent has a better solubility, oral availability, and decreased mean elimination half-life (~18–30 h), leading to early removal of drug from the body compared to the parent compound (rapamycin). Because of the better absorption, it has higher bio-availability (30–60%) and a *T*_max_ of 1–2 h. Everolimus is an immunosuppressive, anti-inflammatory drug that inhibits host cell proliferation by arresting the progression of cell cycle from G1 to S phase; the immune suppressive function is exerted by inhibiting IL-2 and IL-15 mediated lymphocyte proliferation (Kovarik et al., 2002; Lingaraju et al., 2010; Ahya et al., 2011). In addition, everolimus promotes autophagy by inhibiting mTORC1 (Saran et al., 2015).

Inhibition of mTOR pathway by everolimus treatment has been reported to improve cellular immune response in both animal models and human studies. In a study performed with 218 healthy volunteers of >65 years of age, everolimus treatment had beneficial effects over aging-related issues (Mannick et al., 2014). Specifically, these elderly volunteers treated with a low dose of everolimus showed about 20% improvement in their protective response after influenza vaccination. This improvement was associated with reduced expression of programmed death-1 receptor, which is otherwise highly expressed in aging individuals, on CD4 and CD8 T cells, thus increasing T cell antigen processing and expression. This low dose administration (0.5 mg daily or 5 mg weekly) of eveolimus demonstrated minimum number of adverse events, (35 adverse events) compared to a higher dose administration (20 mg weekly), which resulted in 109 adverse events amongst 53 elderly individuals (Mannick et al., 2014). This study clearly highlights the importance of optimizing the dose of mTOR inhibitors, such as everolimus in this case, for better efficacy with minimal adverse effects. In contrast, case studies with organ transplant patients have mentioned a higher risk of Mtb infection and reactivation of LTBI as possible side-effects of everolimus administration (Kovarik et al., 2002; Fijałkowska-Morawska et al., 2011). Although patients in this study were treated with a higher dose of everolimus, than the influenza vaccine study mentioned above, the mechanism underlying the connection between dose of everolimus and reactivation of LTBI is not clearly understood. However, the negative consequences of high dose administration of everolimus can be overcome by co-administration with CYP3A4 enzyme inducers, such as rifampicin, which are used to treat opportunistic TB infections in organ transplant patients (Eisen et al., 2003). Thus, it is important to understand the dose-response of everolimus in the context of host cellular functions and how the drug influences phagocytosis and autophagy-mediated elimination of Mtb during infection.

All the mTOR inhibitors described above are also frequently used in the treatment of various forms of cancer, including breast cancer, renal cell carcinoma and tuberous sclerosis complex, due to their ability to inhibit host cell proliferation and growth (Pohanka, 2006; Koh et al., 2013). However, the idea of using these mTOR inhibitors as potential adjunct HDT for TB therapy needs to be substantiated through experimental evidences related to dosage, pharmacokinetics and pharmacodynamics (PK/PD) parameters and, cost vs. benefit effects on the host immunity. Such evidences need to be reinforced by series of studies on reliable and relevant pre-clinical animal models of Mtb infection. In addition, metabolic dysfunctions, such as hyperglycemia is a common side effect in cancer patients treated with mTOR inhibitors, including everolimus (Porta et al., 2011). Although the impact of such inhibitors in the context of TB treatment remains to be determined, serious side effects of HDT drugs preclude their potential use in any treatment. Moreover, as immune-suppressing agents, the application of mTOR inhibitors as a stand-alone HDT therapy for TB holds a significant risk of reactivation of latent Mtb infection, similar to the situation observed in rheumatoid arthritis patients treated with anti-TNF-α antibody (Kovarik et al., 2002). However, when used at an immune modulating-, as opposed to immune suppressing- dose, these mTOR inhibitors can be potential candidates to serve as an adjunct therapeutic molecule, along with standard anti-TB drugs, in improving the treatment outcome. Thus, fine-tuning the dose of mTOR inhibitors is an important and necessary step toward application of these HDT compounds for TB treatment. Importantly, since Mtb cannot develop resistance to a drug that targets host signaling pathway, such as mTOR or cellular processes, such as autophagy, HDT drugs has the potential to alleviate the development of MDR- and XDR-Mtb strains and their transmission in the community. Analogous to the trend in cancer treatment that have shifted from chemotherapeutic and radiologic regimens to more-host targeted treatment approaches, Mtb infection and/or disease can benefit from specific HDT drugs that targets, for example, the mTOR pathway and/or autophagy.

## Summary and conclusion

Pathogenic Mtb possess several virulent determinants, such as the unusually lipid-rich cell wall, that serve as permeability barriers and protects the bacteria from the harsh intracellular environment within phagocytes, and from the bactericidal activities of anti-TB drugs. Additionally, these mycobacteria-derived molecules interact with the host immune cells and modulates their function, promoting bacterial survival/persistence, causing disease within the host and enabling the development of bacterial drug resistance. Thus, when the innate defensive mechanisms of phagocytes are manipulated by the pathogen to promote its survival, the host develops active disease, which is hard to overcome. This can be one of the reasons for the inefficiency of current anti-mycobacterial drugs to eliminate TB, and for the emergence of drug-resistant Mtb strains. Perturbing host cell functions through HDT molecules has the potential to enhance the effector functions of these cells, which are the ultimate arsenals in combating bacterial infection. Moreover, these immune modulating drugs do not contribute to the emergence of drug resistance by the infecting bacteria. This criterion is crucial when considering therapy, particularly for patients with MDR, XDR-TB, as well as those patients with co-existing chronic conditions, such as diabetes or HIV infection, in which conventional antibiotics therapy has been shown to be complex, complicated, toxic and insufficient in achieving a bacteriological cure. Host cell autophagy, regulated by mTOR pathway, plays an important role in cellular homeostasis as well as in antibacterial defense mechanism. Therefore, targeting mTOR pathway with small molecules, such as everolimus, has the potential to develop novel and better combination drug therapy, along with standard anti-TB drugs to combat various forms of TB in patients with/without other co-morbid conditions. This approach can also enhance bacterial killing, reduce treatment duration, and/or improve clinical outcome. Clearly, more research and experimental evidence is warranted on these and other HDT molecules, for their efficacy, toxicity and other properties, through extensive pre-clinical studies using appropriate animal models of TB, before they are tried as therapeutic intervention for TB in human clinical trial.

## Author contributions

SS: conceived the concept; PS and SS: wrote/edited the manuscript and agreed for submission.

### Conflict of interest statement

The authors declare that the research was conducted in the absence of any commercial or financial relationships that could be construed as a potential conflict of interest.
